# NDPACX: a newly defined X-linked Parkinsonian syndrome associated with *SLC9A6* hemizygote mutation

**DOI:** 10.1093/braincomms/fcaf435

**Published:** 2025-11-05

**Authors:** Ryotaro Okochi, Yoshihiro Nihei, Daisuke Ito

**Affiliations:** Department of Neurology, Keio University School of Medicine, Tokyo 160-8582, Japan; Department of Neurology, Keio University School of Medicine, Tokyo 160-8582, Japan; Department of Neurology, Keio University School of Medicine, Tokyo 160-8582, Japan; Memory Center, Keio University School of Medicine, Tokyo 160-8582, Japan

**Keywords:** SLC9A6, NDPACX, Christianson syndrome, Parkinson's disease, lysosome

## Abstract

X-linked female-restricted neurodegenerative disorder with Parkinsonian syndrome and cognitive impairment (NDPACX) is a recently recognized clinical entity caused by heterozygous mutations in *SLC9A6* on the X chromosome, which encodes the endosomal Na⁺/H⁺ exchanger NHE6 that contributes to endolysosomal trafficking and acidification. While hemizygous *SLC9A6* mutations in males cause Christianson syndrome, a severe neurodevelopmental disorder, emerging evidence indicates that female carriers may develop progressive, adult-onset Parkinsonian syndrome variably accompanied by cognitive decline and psychiatric symptoms. These features often resemble those of corticobasal degeneration, progressive supranuclear palsy, and atypical Parkinson’s disease. Although pathological data for NDPACX are currently lacking, mechanistic inferences drawn from Christianson syndrome and animal models implicate endosomal dysfunction, impaired receptor recycling, and tau accumulation, depending on the loss of function. Furthermore, recent findings have linked reduced *SLC9A6* expression to the increased vulnerability of the substantia nigra in sporadic Parkinson’s disease, suggesting a broader relevance beyond rare monogenic disorders. In particular, NDPACX represents a model of endolysosomal neurodegeneration that may be related to lysosomal storage diseases or autophagy-related mechanisms commonly implicated in Parkinsonian syndromes. In this review, we summarize the clinical, molecular, and emerging pathological insights into NDPACX and propose that targeting endolysosomal homeostasis may open new therapeutic avenues for both hereditary and idiopathic neurodegenerative diseases characterized by proteinopathies.

## Teaser

Okochi R. *et al*. summarize the clinical, pathological, and molecular features of Neurodegenerative Disorder, X-linked, Female-Restricted, with Parkinsonism and Cognitive impairment, NDPACX, a recently defined entity caused by heterozygous SLC9A6 mutations. They highlight its clinical features, including parkinsonian syndrome and propose endolysosomal dysfunction as a potential therapeutic target.

## Graphical Abstract

**Graphical Abstract:**
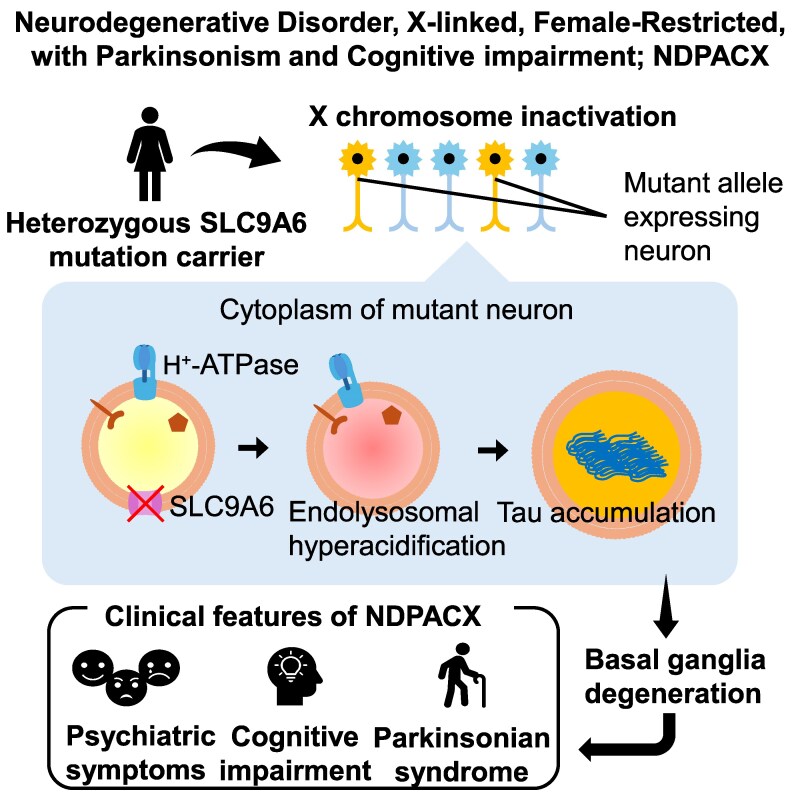

## Introduction

Christianson syndrome, formally classified as mental retardation, X-linked syndromic Christianson type (MRXSCH), is a severe neurodevelopmental disorder characterized by microcephaly, impaired ocular movements, progressive global developmental delay, hypotonia, developmental regression, abnormal involuntary movements, and various early-onset seizures. This disorder, first described by Christianson *et al*. in 1999,^1^ is caused by pathogenic variants of the *SLC9A6* gene located on the X chromosome. In hemizygous males, mutations in *SLC9A6* lead to the classical MRXSCH phenotype, which partially overlaps with the clinical presentation of Angelman syndrome. Reported pathogenic variants are predominantly nonsense or missense point mutations resulting in loss of protein function, although small insertions/deletions and splice-site variants have also been described. In most affected families, the disorder is maternally inherited via carrier females, but de novo mutations have also been reported.^1-6^

Traditionally, female carriers of *SLC9A6* mutations have been considered asymptomatic or mildly affected by intellectual disabilities and psychiatric disorders. However, recent reports, including those from our own group, have identified female carriers presenting with progressive Parkinsonian syndrome, challenging the earlier understanding of the disease spectrum.^7-11^ In response to this emerging phenotype, a new clinical entity, an X-linked female-restricted neurodegenerative disorder with Parkinsonian syndrome and cognitive impairment (NDPACX), was registered in the Online Mendelian Inheritance in Man database in March 2025.

NDPACX is characterized by adult-onset progressive movement disorders consistent with the Parkinsonian syndrome, variably accompanied by cognitive impairment and psychiatric symptoms. The clinical features often mimic those of other neurodegenerative disorders, such as progressive supranuclear palsy (PSP), corticobasal degeneration (CBD), and clinically established Parkinson’s disease; however, the severity varies, ranging from near-health to severe disability. The identification of NDPACX underscores the intriguing possibility that *SLC9A6*, previously associated with neurodevelopmental pathology, plays a critical role in adult-onset neurodegeneration.

*SLC9A6* encodes a Na⁺/H⁺ exchanger localized to early endosomes that is essential for maintaining endosomal-lysosomal homeostasis.^7,12^ Growing evidence implicates haploinsufficiency of lysosome-related genes as a common mechanism in neurodegenerative diseases.^8-11^ NDPACX appears to exemplify this category and represents a novel form of neurodegeneration related to endosomal-lysosomal dysfunction. Notably, female carriers develop a broad spectrum of symptoms, and this incomplete penetrance suggests that X-chromosome inactivation (XCI) serves as a key modifier of disease expression, adding further complexity to its pathophysiology.

In this review, we provide an overview of the current understanding of NDPACX, including its clinical phenotype, pathological features, and the molecular and cellular functions of SLC9A6. We also discuss the role of XCI in modulating disease manifestations and explore how insights into endosomal and lysosomal dysfunction may inform future therapeutic strategies for neurodegenerative diseases.

## Molecular mechanism of SLC9A6

SLC9A6, also known as Na⁺/H⁺ exchanger isoform 6 (NHE6), is a member of the SLC9A subfamily, which belongs to the broader solute carrier superfamily.^13-15^ This family comprises nine known isoforms (NHE1–NHE9), which can be broadly classified into plasma membrane isoforms (NHE1–NHE5) and organellar isoforms (NHE6–NHE9) based on phylogenetic and structural analyses.^16^ Among the organellar subtypes, NHE6, NHE7, and NHE9 share high sequence homology, whereas NHE8 is structurally distinct and possesses a notably shorter and divergent C-terminal hydrophilic domain, suggesting that it may have unique regulatory functions.^16^

SLC9A6 predominantly localizes to early and recycling endosomes, where it regulates luminal pH by exchanging intravesicular protons for cytosolic sodium ions.^7^ Notably, it does not co-localize with lysosomal markers, the trans-Golgi network, or mitochondria, although it can be transiently detected at the plasma membrane.^7^ Within endosomal compartments, SLC9A6 primarily overlaps with early endosomal antigen 1 and exhibits a distinct yet complementary distribution relative to NHE9, which is enriched in later stages of the recycling endosomal pathway.^12^ This spatial organization supports a model in which NHE isoforms function coordinately to regulate pH and ion gradients throughout the endosomal system.

**Figure 1:**
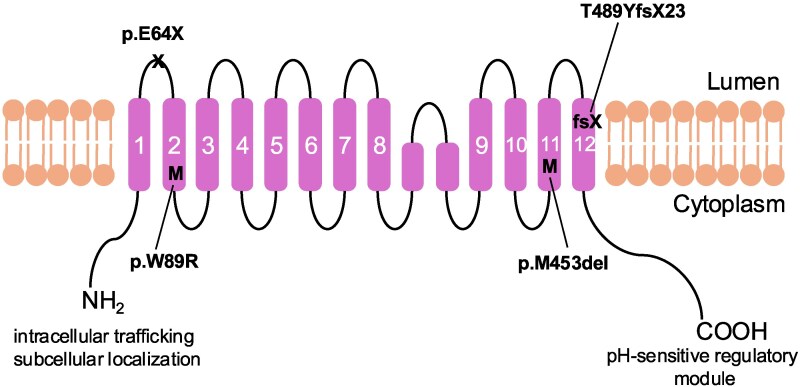
**Structure of SLC9A6.** Schematic illustration of the human SLC9A6 protein (NM_001042537). SLC9A6 consists of approximately 650 amino acids and forms 12 transmembrane helices, predicted by TMHMM-2.0, embedded in the endosomal membrane. Both the N- and C-terminal hydrophilic domains are cytoplasmic. The N-terminal domain contains motifs involved in intracellular trafficking and subcellular localization. The C-terminal domain functions as a pH-sensitive regulatory module that modulates exchanger activity in response to cytoplasmic acidification. These features are conserved across homologous Na^+^/H^+^ exchangers in yeast and plants, highlighting the evolutionary conservation of endosomal pH regulation. Mutations related to Parkinsonian syndromes have also been identified. M: missense; X: premature stop; fs: frameshift.

Structurally, SLC9A6 comprises approximately 650 amino acids and exhibits the characteristic 12-transmembrane domain topology of the Na⁺/H⁺ exchanger family^16,17^ (Fig. 1). These membrane-spanning segments form the ion-conducting pore embedded in the lipid bilayer of endosomal membranes. Both N-terminal and C-terminal hydrophilic domains face the cytoplasm and are crucial for regulating transporter localization, activity, and protein–protein interactions. The subcellular localization of SLC9A6 appears to be determined by specific structural motifs within the cytoplasmic N-terminal domain and internal loops, which likely mediate recognition by trafficking machinery. Conversely, the C-terminal domain functions as a pH-sensitive regulatory module that modulates exchanger activity in response to cytoplasmic acidification. These topological features are consistent with structural models of homologous transporters in yeast and plants, underscoring the evolutionary conservation of endosomal pH regulation.^12^

**Figure 2:**
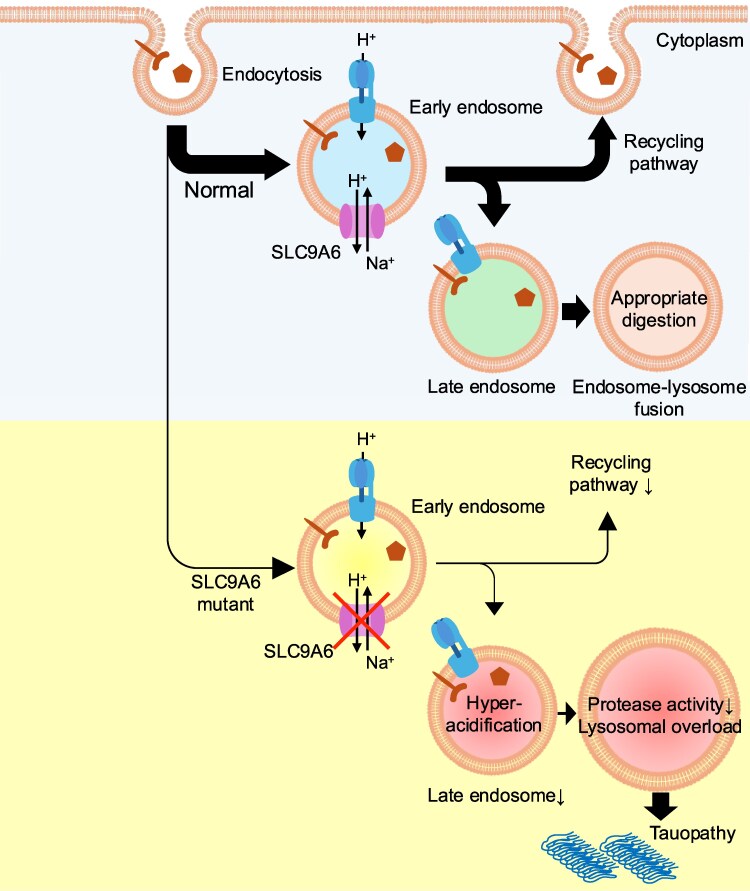
**Endolysosomal system and function of SLC9A6.** The upper panel shows the normal endolysosomal system with functional SLC9A6. A proper balance between proton influx via vacuolar-type H^+^-ATPase and proton efflux via SLC9A6 maintains optimal pH in early endosomes. This pH homeostasis supports efficient sorting between the recycling and lysosomal pathways, ensuring appropriate receptor trafficking and surface membrane maintenance. The lower panel shows the pathological state of SLC9A6 dysfunction. Loss of SLC9A6 function leads to overacidification of early endosomes, which impairs endocytosis and reduces recycling. Acidification also disrupts lysosomal protease activity and reduces endosome–lysosome fusion, resulting in lysosomal pathway overload and impaired degradative capacity.

SLC9A6 plays an essential role in neuronal physiology, particularly in maintaining endosomal pH homeostasis, crucial for receptor recycling, neurotrophin signalling, and intracellular trafficking of membrane proteins^12,18^ (Fig. 2). A key example is the recycling of the TrkB receptor and its associated brain-derived neurotrophic factor signalling, which are vital for neuronal survival and plasticity.^18^ Loss of SLC9A6 function leads to endosomal overacidification, disrupting these signalling pathways and impairing synaptic development and function. Furthermore, the endosomal system participates in dendritic spine growth and remodelling during long-term potentiation, a cellular substrate of learning and memory.^19^ Consequently, dysregulation of endosomal pH caused by SLC9A6 deficiency may broadly affect neuronal circuit formation and synaptic regulation.

Consistent with these findings, neurological consequences of *SLC9A6* mutations appear to arise from a combination of impaired membrane trafficking, defective receptor recycling, and endosomal dysfunction. In hemizygous males, such mutations lead to neurodevelopmental disorders, including Christianson syndrome. Conversely, in heterozygous females, they may cause late-onset neurodegenerative phenotypes and behavioural abnormalities such as NDPACX. In the latter, the mechanism likely involves haploinsufficiency, which is potentially modified by skewed XCI. This dosage sensitivity places *SLC9A6* among a growing number of genes in which mosaic loss-of-function disrupts endolysosomal homeostasis, ultimately resulting in the dysfunction of protein quality control in progressive neurological diseases.^16,17^

## Neuropathological features and tau accumulation in NDPACX

Postmortem analyses of individuals with *SLC9A6*-related disorders have revealed consistent patterns of neurodegeneration affecting deep brain structures and the cerebellum. In affected males with Christianson syndrome, severe neuronal loss and gliosis occur in the globus pallidus, putamen, substantia nigra, and cerebellar cortex, with less prominent involvement of the caudate nucleus, pontine nuclei, inferior olives, and cerebellar dentate nucleus. The cerebral cortex and hippocampus remain preserved. Despite this preservation, diffuse glial tau pathology has been detected in white matter tracts of the centrum semiovale, cerebellum, and brainstem. Microscopic examination reveals widespread tau-positive glial inclusions, including coiled structures throughout the white matter and neurofibrillary tangle-like formations within neurons of the basal ganglia, thalamus, and midbrain nuclei. Tau-positive astrocytic plaques appear in the cerebral white matter, thalamus, and brainstem. These changes represent characteristic features of corticobasal degeneration, though without other histopathological hallmarks of CBD or ballooned neurons. Pathological tau predominantly comprises 4-repeat (4R) isoforms, with ultrastructural features resembling both paired helical and ribbon-like filaments.^4^ Importantly, co-pathologies typically seen in other neurodegenerative diseases, including amyloid-deposit, α-synuclein, and TDP-43 positive inclusions, have not been observed.^20^ This absence suggests that SLC9A6-related disorders may predominantly involve tau pathology without evidence of additional proteinopathies.^4^

In female carriers of *SLC9A6* mutations diagnosed with NDPACX, focal tau accumulation has been demonstrated using positron emission tomography (PET) imaging with selective tracers such as ¹⁸F-florzolotau. The striatum and basal ganglia frequently show asymmetric signal intensities that correlate with the lateralization and severity of extrapyramidal symptoms.^21^ These findings reinforce the view that tauopathy plays a mechanistic role in the extrapyramidal phenotype of symptomatic female carriers. Notably, postmortem studies in this population remain limited, necessitating further histopathological validation.

Mouse models have provided critical insights into the pathophysiology of SLC9A6 deficiency. In a foundational study, Strømme *et al*. demonstrated that Slc9a6 knockout mice display endosomal-lysosomal dysfunction with features reminiscent of lysosomal storage disorders. These include abnormal accumulation of GM2 ganglioside and unesterified cholesterol within late endosomes and lysosomes of neurons in vulnerable brain regions such as the hippocampus, amygdala, and cerebral cortex. Additionally, the mice exhibit progressive Purkinje cell loss in the cerebellum, neuroaxonal dystrophy, and behavioural abnormalities, including hyperactivity and cerebellar signs.^22^ Recently, NHE6-deficient rats have shown age-related tau deposition with widespread distribution, such as cortical and subcortical regions, recapitulating tau pathology dependent on SLC9A6 loss of function.^23^ These findings underscore the importance of SLC9A6 in endosomal membrane trafficking, lipid homeostasis, and protein quality control, particularly in neurons.

Building on this understanding, Sikora *et al*. investigated heterozygous female Slc9a6 knockout mice, modelling the genetic mosaicism observed in female carriers of Christianson syndrome.^24^ These animals exhibit mosaic neuropathological changes, including gliosis and regional neurodegeneration, particularly in the cerebellum. Moreover, they display behavioural abnormalities such as impaired visuospatial memory, reduced social interaction, and increased anxiety-like behaviour. This study established that mosaic loss of *SLC9A6* due to XCI is sufficient to produce significant neurodevelopmental and neurodegenerative phenotypes, reinforcing the disease relevance of *SLC9A6* haploinsufficiency in female NDPACX carriers.

Collectively, human and animal data converge on a neuropathological profile characterized by tau accumulation, selective neuronal loss, and endosomal-lysosomal dysfunction. The enrichment of 4R tau isoforms and the spatial pattern of degeneration further suggest a mechanistic overlap with primary tauopathies such as corticobasal degeneration and progressive supranuclear palsy. These findings indicate that therapeutic strategies targeting tau aggregation or restoring endolysosomal homeostasis may be applicable for NDPACX-and related SLC9A6-linked disorders.

## Lysosomal dysfunction and neurodegenerative proteinopathies

Growing evidence implicates endolysosomal dysfunction as a key driver of neurodegeneration, including Parkinsonian syndromes. Beyond rare monogenic lysosomal storage disorders (LSDs), lysosomal gene dysfunction is increasingly recognized in common neurodegenerative proteinopathies, including Alzheimer's disease (AD), Parkinson's disease (PD), frontotemporal dementia (FTD), and amyotrophic lateral sclerosis.^11,25^ Prominent examples include GBA1, SMPD1, and GALC, for which heterozygous variants are associated with elevated risk of developing PD.^9,25,26^ Recently, arylsulfatase A, which causes metachromatic leukodystrophy, an autosomal recessive lysosomal storage disease, has been reported as a genetic modifier of Parkinson's disease, contributing to α-synuclein homeostasis as a chaperone.^27^ Additionally, PD-associated genes such as *LRRK2*, *VPS35*, *ATP13A2*, *PINK1*, and *PRKN* regulate endolysosomal and autophagic dynamics,^28-32^ further supporting the view that this cellular system represents a common vulnerability point in Parkinsonian syndromes. Autosomal-recessive LSD Niemann-Pick type C1, which results in intracellular accumulation of cholesterol and lipids in the endosomal/lysosomal network, is classified as a tauopathy due to the presence of intracellular tau aggregates that are biochemically identical to those found in AD.^33^ In FTD, lysosomal dysfunction resulting from genetic mutations (e.g. progranulin, TMEM106B) leads to aggregation of TDP-43, constituting a key pathogenic mechanism.^34^ PGRN haploinsufficiency impairs lysosomal homeostasis, causing defective hydrolase trafficking and reduced enzymatic degradation, which in turn promotes the accumulation of phosphorylated TDP-43. Elevated TMEM106B expression associated with risk alleles disrupts lysosomal acidification and transport, contributing to lysosomal functional impairment. Collectively, these mechanisms drive TDP-43 accumulation and neurodegeneration in patients with FTD.

In this context, NDPACX (X-linked female-restricted neurodegenerative disorder with Parkinsonian syndrome and cognitive impairment) has emerged as an important disease model linking endosomal dysfunction specifically to Parkinsonian features. NDPACX results from heterozygous mutations in *SLC9A6*, which encodes an early and recycling endosome-resident sodium-hydrogen exchanger (Fig. 2). Notably, the pathological mechanism of NDPACX appears to be endosome-specific and distinct from generalized lysosomal storage or autophagic failure. This unique feature highlights the importance of endosomal pH regulation, membrane trafficking, cargo sorting, and protein quality control in maintaining dopaminergic neuronal integrity.^23^

Notably, the role of SLC9A6 extends beyond rare inherited disorders. In a recent transcriptomic analysis of postmortem brain samples, Prasad *et al*. reported significantly reduced SLC9A6 expression in the substantia nigra of individuals with sporadic Parkinson's disease.^35^ This observation suggests that decreased SLC9A6 activity may contribute to nigral vulnerability in both familial and idiopathic PD, likely through dysregulation of vesicular pH homeostasis and impaired endosomal recycling of key synaptic and trophic components. This study further supports the notion that SLC9A6 dysfunction may represent a broader, underrecognized mechanism underlying Parkinsonian syndromes.

Collectively, these data underscore the importance of maintaining endosomal-lysosomal homeostasis for neuronal survival. The study of NDPACX provides a genetically and mechanistically defined model of endolysosome-specific Parkinsonian neurodegeneration and offers critical insights into the broader field of Parkinsonian syndromes. As lysosomal-enhancing and autophagy-targeting therapies advance towards clinical translation, the inclusion of endolysosome-focused strategies, such as those addressing SLC9A6 dysfunction, may prove essential for developing more effective interventions for both rare and common Parkinsonian syndromes.

## Female carriers and the clinical spectrum of NDPACX

While male individuals with *SLC9A6* mutations consistently develop Christianson syndrome, typically presenting with seizures before the age of 2 years along with developmental delay and ataxia,^5^ the clinical picture of heterozygous female carriers is highly variable and has only recently been appreciated in full scope. Earlier studies regarded most female carriers as asymptomatic or minimally affected. However, emerging evidence has revealed a broad neuropsychiatric and neurodegenerative phenotype in a significant proportion of carriers, ranging from developmental and cognitive deficits from birth to adult-onset Parkinsonian syndrome, most often manifesting between the fifth and eighth decades of life (Table 1; see also Supplementary Table 1 for the complete dataset).

**Table 1:** Neurological findings in reported cases of NDPACX in the literature

No.	Reference	Mutation	Onset age of parkinsonism	Type of parkinsonism	L-dopa reactivity	Intellectual disability	Cognitive function	Psychiatric symptoms	MIBG	DAT-SPECT	MRI
1	Riess *et al*. (2013)^3^	c.1464_1465insT p.Thr489TyrfsX23	Mid 50s	Parkinson's disease	N/A	N/A	N/A	Depression	N/A	N/A	Slight general brain atrophy
2	Riess *et al*. (2013)^3^	c.1464_1465insT p.Thr489TyrfsX23	70s	Parkinsonian syndrome	N/A	N/A	N/A	N/A	N/A	N/A	N/A
3	Sinajon *et al*. (2016)^36^	c.190G>T p.E64X	Early 60s	CBD	N/A	Moderate	MMSE: 22/30 Behavior Neurology Assessment-Short Form: 48/114	Emotional lability	N/A	N/A	Atrophy in left frontal and parietal cortex
4	Pescosolid *et al*. (2019)^2^	N/A	Mid 60s	CBD	N/A	Severe	Rapid cognitive decline	N/A	N/A	N/A	Atrophy in left frontal and parietal cortex
5	Pescosolid *et al*. (2019)^2^	N/A	Early 50s	Parkinsonian syndrome	N/A	Mild	MMSE: 27/30	N/A	N/A	N/A	Generalized brain atrophy and cerebellar atrophy
6	Nan *et al*. (2022)^20^	c. 265T>C p.Trp89Arg	Late 40s	Atypical parkinsonian syndrome	No	Severe	Full scale IQ: 42 (JWAIS-Ⅲ)	No	Normal	Reduced (SBR: 3.98/3.16)	Normal
7	Nan *et al*. (2022)^20^	c. 265T>C p.Trp89Arg	Mid 60s	Atypical parkinsonian syndrome	No	Severe	Progressive cognitive decline	No	Normal	Reduced (SBR: 2.16/1.20)	Atrophy in left superior parietal cortex
8	Nan *et al*. (2022)^20^	c. 265T>C p.Trp89Arg	N/A	Parkinsonian syndrome	N/A	Mild	Full scale IQ: 68 (JWAIS-Ⅲ)	No	N/A	N/A	N/A
9	Yamamoto *et al*. (2025)^21^	c, 1357_1359del p.Met453del	Early 40s	Parkinson's disease	Yes	N/A	Full scale IQ: 64 (JWAIS-Ⅲ)	Anxiety	Normal	Reduced (SBR: 3.47/4.76)	N/A

CBD, Corticobasal Degeneration; DAT-SPECT, Dopamine Transporter Single Photon Emission Computed Tomography; IQ, Intelligence Quotient; JWAIS-III, Japanese version of the Wechsler Adult Intelligence Scale, Third Edition; L-dopa, Levodopa; MIBG, Metaiodobenzylguanidine scintigraphy; MMSE, Mini-Mental State Examination; MRI, Magnetic Resonance Imaging; N/A, Not Available/Not Applicable; SBR, Specific Binding Ratio. Note: A full version of this table with all data is provided in Supplementary Table 1.

In a seminal report by Sinajon *et al*. (2016),^36^ four female carriers of an *SLC9A6* nonsense mutation (E64X) exhibited various psychiatric and behavioural disorders, including learning difficulties, self-injurious behaviour, tantrums, aggression, and psychiatric diagnoses such as depression, anxiety, psychosis, and possible schizophrenia. Symptoms often worsened across generations, suggesting a genetic anticipation-like phenomenon or the influence of modifying factors. One older female in the same family developed a progressive CBD-like neurodegenerative syndrome with akinetic-rigid features, supranuclear gaze palsy, myoclonus, and cortical atrophy, culminating in death from aspiration pneumonia at age 64.

A neuropsychological study of 20 heterozygous females by Pescosolido *et al*. (2019)^2^ revealed that 85% exhibited deficits in at least one cognitive domain, with impairments in visuospatial function, attention, and executive function being most common. Approximately one-third had intelligent quotient (IQ) scores below 70, and psychiatric or behavioural diagnoses (attention-deficit hyperactivity disorder, autism spectrum disorder, and schizoaffective disorder) were prevalent. Importantly, two older carriers developed CBD and atypical Parkinsonian syndrome, reinforcing the potential link between *SLC9A6* and adult-onset tauopathies.

Nan *et al*. (2022)^20^ and Yamamoto *et al*. (2025)^21^ further characterized a Japanese family in which three women exhibited varying degrees of Parkinsonian syndrome. They developed Parkinsonian features and exhibited tau accumulation in the striatum on ¹⁸F-florzolotau tau PET imaging. ¹¹C-Pizzburgh compound B amyloid PET results were negative in all patients. One patient responded to L-dopa (meeting criteria for clinically established Parkinson's disease) with normal ¹²³I-metaiodobenzylguanidine scintigraphy uptake,^37^ while others were classified as having PSP-like atypical Parkinsonian syndrome. Biomarker analysis revealed elevated GFAP and decreased neurofilament light chain in affected carriers. Notably, asymmetric tau tracer accumulation in the striatum, contralateral to extrapyramidal symptoms, correlated with severity, indicating that NDPACX is a novel tauopathy. Conversely, all subjects in our study had non-progressive intellectual disabilities from early childhood without tau accumulation in the cortex. This pattern of congenital cognitive impairment suggests a developmental origin, likely due to endosomal-lysosomal dysfunction caused by *SLC9A6* mutations, rather than tau accumulation.

Multiple reports have documented learning disabilities, language and reading impairments, executive dysfunction, and mild-to-moderate intellectual disabilities in female carriers. In several families (e.g. Masurel-Paulet *et al*., 2016^38^; Schroer *et al*., 2010^5^), female carriers exhibit childhood-onset speech disorders, dyslexia, and dissociation between verbal and performance IQ, often requiring speech therapy and educational interventions.

Female carriers and their clinical spectrum have been increasingly recognized, but the population frequency of pathogenic SLC9A6 variants among carriers remains extremely low. According to ClinVar (NCBI, https://www.ncbi.nlm.nih.gov/clinvar/), we focused on variants classified as pathogenic or likely pathogenic. Although more than fifty variants have been reported in ClinVar, genetic ancestry group frequencies remain unclear. Only two—chrX:135,985,560C>T (NM_001379110.1:c.-56-43C>T) and chrX:136,012,948C>T (NM_001379110.1:c.886-1C>T)—have been detected in large population databases, specifically in African and European cohorts. In gnomAD v4.1.0 (https://gnomad.broadinstitute.org/), their combined carrier frequency was about 0.0012% globally (0.007% in Africans, 0.0011% in Europeans). In TOPMed freeze 10 (https://bravo.sph.umich.edu), the frequency was slightly higher at 0.0020% globally (0.015% in Africans, 0.0011% in Europeans). These findings suggest a relatively higher frequency in Africans, although this difference may reflect stochastic variation from limited sampling rather than a true population-specific effect.

Interestingly, not all carriers are symptomatic. Some families include asymptomatic carriers or females with minimal features despite confirmed mutations.^1,6^ Analysis of XCI patterns in blood or lymphocytes has yielded inconsistent results, suggesting that brain-specific or mosaic XCI patterns may serve as modifiers of penetrance and phenotype.

## Conclusion

The identification of NDPACX, an X-linked neurodegenerative disorder manifesting as Parkinsonian syndrome and cognitive impairment in female carriers of *SLC9A6* mutations, challenges the conventional dichotomy between neurodevelopmental and neurodegenerative disease paradigms. Originally described as the genetic cause of Christianson syndrome in hemizygous males,^1,6^  *SLC9A6* has now emerged as a critical gene involved in endosomal homeostasis and neuronal resilience throughout the lifespan.

Mechanistic studies have highlighted the essential role of SLC9A6 in regulating endosomal pH and receptor trafficking, which are processes fundamental to synaptic plasticity, neurotrophin signalling, and membrane recycling.^16,18,19^ Both human neuropathology and animal models converge on a shared pathophysiological cascade: endolysosomal dysfunction, disrupted protein trafficking, and selective neuronal vulnerability, often accompanied by a dysfunction in protein quality control and tau accumulation.^4,22,23^ These features align NDPACX with a broader spectrum of 4R tauopathies and point to common pathways shared by corticobasal degeneration and progressive supranuclear palsy.

Risk variants of lysosomal genes can impair enzymatic activity and substrate degradation, leading to the accumulation of toxic proteins and proteinopathies. Accordingly, lysosomal pathways and their molecular components are emerging as promising pharmacological targets.^39^ Enzyme replacement therapy, successfully applied in Gaucher disease with recombinant glucocerebrosidase, involves administering functional enzymes to restore lysosomal degradation capacity.^40,41^ Additionally, clinical trials of adeno-associated viral vector-based gene therapy for patients with Hurler syndrome (Mucopolysaccharidosis Type I, or MPS I), a neurotypical lysosomal storage disorder, are currently ongoing (NCT03580083). This therapy aims to deliver a functional copy of the α-L-iduronidase gene to the central nervous system. These efforts seek to translate similar strategies to both NDPACX and a broader class of endolysosome-driven neurodegenerative disorders. Furthermore, the unique model of X-linked hemizygosity and mosaicism in NDPACX offers a valuable lens through which to study disease modifiers such as X-chromosome inactivation, with implications extending beyond this specific syndrome.

Neurologists should recognize this condition when evaluating patients with movement disorders in clinical practice. Continued investigation into the molecular and cellular underpinnings of SLC9A6 function will not only deepen our understanding of NDPACX but may also provide critical insights into converging mechanisms underlying Parkinsonian syndromes and tauopathies in general. At present, clinical genetic testing for SLC9A6 mutations is available through multigene panels for epilepsy and neurodevelopmental disorders or next-generation sequencing platforms in many developed countries, but access remains limited in low-resource settings. Wider implementation and accessibility of such testing will be essential for accurate diagnosis, appropriate genetic counseling, and future therapeutic trials.

## Supplementary Material

fcaf435_Supplementary_Data

## Data Availability

Data sharing is not applicable to this article as no new data were created or analysed in this study.
